# Acromegaly Caused by Ectopic Growth Hormone Releasing Hormone Secretion: A Review

**DOI:** 10.3389/fendo.2022.867965

**Published:** 2022-06-09

**Authors:** Iga Zendran, Gabriela Gut, Marcin Kałużny, Katarzyna Zawadzka, Marek Bolanowski

**Affiliations:** ^1^ Department of Endocrinology, Diabetes and Isotope Therapy, Wroclaw Medical University, Wroclaw, Poland; ^2^ Department of Endocrinology, Diabetes and Isotope Therapy, Students research association, Wroclaw Medical University, Wroclaw, Poland

**Keywords:** acromegaly, ectopic, GHRH, neuroendocrine tumors, pituitary hyperplasia

## Abstract

**Introduction:**

Ectopic acromegaly is a rare condition caused most frequently by growth hormone releasing hormone (GHRH) secretion from neuroendocrine tumors. The diagnosis is often difficult to establish as its main symptoms do not differ from those of acromegaly of pituitary origin.

**Objectives:**

To determine most common clinical features and diagnostic challenges in ectopic acromegaly.

**Patients and Methods:**

A search for ectopic acromegaly cases available in literature was performed using PubMed, Cochrane, and MEDline database. In this article, 127 cases of ectopic acromegaly described after GHRH isolation in 1982 are comprehensively reviewed, along with a summary of current state of knowledge on its clinical features, diagnostic methods, and treatment modalities. The most important data were compiled and compared in the tables.

**Results:**

Neuroendocrine tumors were confirmed in 119 out of 121 patients with histopathological evaluation, mostly of lung and pancreatic origin. Clinical manifestation comprise symptoms associated with pituitary hyperplasia, such as headache or visual field disturbances, as well as typical signs of acromegaly. Other endocrinopathies may also be present depending on the tumor type. Definitive diagnosis of ectopic acromegaly requires confirmation of GHRH secretion from a tumor using either histopathological methods or GHRH plasma concentration assessment. Hormonal evaluation was available for 84 patients (66%) and histopathological confirmation for 99 cases (78%). Complete tumor resection was the main treatment method for most patients as it is a treatment of choice due to its highest effectiveness. When not feasible, somatostatin receptor ligands (SRL) therapy is the preferred treatment option. Prognosis is relatively favorable for neuroendocrine GHRH-secreting tumors with high survival rate.

**Conclusion:**

Although ectopic acromegaly remains a rare disease, one should be aware of it as a possible differential diagnosis in patients presenting with additional symptoms or those not responding to classic treatment of acromegaly.

## Introduction

Acromegaly is a rare disease with prevalence ranging between 2.8 and 13.7/100,000 persons, mostly caused by a benign pituitary growth hormone (GH) secreting adenoma (1). The term ‘ectopic acromegaly’ refers to a syndrome caused by secretion of growth hormone releasing hormone (GHRH), or occasionally, by an extra-pituitary source of GH and accounts for less than 1% of all acromegaly cases (2, 3). Ectopic GHRH derives most commonly from functional neuroendocrine tumors (NETs) originating in the lung or the pancreas and results in pituitary hyperplasia and excess GH secretion (4). The term ‘ectopic’ is used in a broader sense in this review, applying not only to its most common meaning of an abnormal localization, but also basically to the secretion of a hormone by a tissue that does not produce it under physiological circumstances (5). Therefore, GHRH-secreting pituitary gangliocytoma will also be included in the review, as they fall under given definition (6). Ectopic acromegaly clinical features are indistinguishable from those of acromegaly caused by a pituitary somatotropinoma (7). The suspicion of ectopic acromegaly is most commonly drawn when no discrete adenoma is shown in magnetic resonance (MR) imaging of the pituitary gland. Additional manifestations of an extracranial tumor, such as cough or dyspnea for the lung neoplasm or other endocrinopathies for a pancreatic NET, as well as lack of remission of the disease after transsphenoidal adenomectomy may also be suggestive of the disease (3, 8).

## Patients and Methods

An extensive search was performed for ectopic acromegaly cases described in literature between 1982 and 2021. Research was conducted in PubMed, Cochrane, and MEDline databases using the particular keywords acromegaly, GHRH, ectopic, and neuroendocrine tumors. More than 300 articles were screened. Duplicates, unusable records, and those with partial information were rejected. According to the specific criteria, 127 cases were selected containing confirmation of ectopic GHRH secretion by biochemical and/or histopathological examination, some of them described as a part of case series. GHRH-secreting pituitary gangliocytoma was also included.

### Historical Aspects

Since the initial report of the possible association between the neuroendocrine functional tumor and acromegaly, suggested by Altmann et al. in 1959, around 170 cases of suspected ectopic acromegaly have been described in literature to date, mostly as case reports (4, 6, 9–21). To our best knowledge, only 19 cases were described as caused by an ectopic source of GH (22–27). Consequently, the vast majority of the cases were reports of acromegaly due to ectopic GHRH secretion. However, until 1982, when the isolation of GHRH from pancreatic tumors was achieved simultaneously by two research groups, the underlying cause of ectopic acromegaly could only have been suspected (28, 29). To date, 10 cases of possible GHRH secretion by neuroendocrine neoplasms have been reported (7 of the lung, 2 of the pancreas, and one of the foregut origin) and will not be covered in this review (4). Also, 13 cases described between 1984 and 2002 and mentioned in previous reviews will also not be included here due to the lack of records. In this review, 127 cases of acromegaly caused by ectopic GHRH secretion confirmed by biochemical or histopathological examination will be discussed, comprehensively summarizing current knowledge on this rare condition.

### Clinical Features

Acromegaly due to ectopic GHRH secretion is more common in women who represent 70% of reported cases (**Table 1**). Patients ranged in age from 14 to 77, with mean age of 43.3 years at the time of diagnosis. The age distribution was comparable for men and women, with mean age of 43.9 and 41.7 years, respectively. The duration of the disease before diagnosis was approximately 7.4 years, which is consistent with the course of acromegaly caused by somatotropinoma (30, 31). Although the age at diagnosis was similar for both men and women, there has been a sex disparity from the time of the onset of symptoms until diagnosis. The diagnostic delay was longer in men by approximately 3.5 years with mean duration of 9.9 years in comparison to 6.2 years in women, which is also in line with pituitary acromegaly characteristics (32).

**Table 1:** Clinical features of 127 patients with GHRH-secreting tumors.

	*n*	%
Female (F)	89	70.1%
Male (M)	38	29.9%
	**Mean ± SD**	**Median (range)**
Age (years) (*n=127*)	43.25 ± 14.7	42 (14-77)
Age F (years) (*n=89*)	43.9 ± 14.6	43 (15-77)
Age M (years) (*n=38*)	41.65 ± 15.1	41.5 (14-74)
Duration (years) (*n=68*)	7.4 ± 5.5	6 (1-22)
Duration F (years) (*n=46*)	6.2 ± 4.3	5 (1-22)
Duration M (years) (*n=22*)	9.9 ± 6.8	8.5 (2-20)

### Tumors Characteristics

Extracranial tumors constituted 78.7% of the cases and the remaining 27 cases presented with GHRH-secreting tumors within the sellar region are shown in **Table 2**. Histopathological evaluation was available for 121 tumors, indicating that 119 of them were neuroendocrine tumors with only 2 exceptions for a pituitary diffuse large B-cell lymphoma (DLBCL) and an adenoid cystic carcinoma of the lung (18, 33). Most common histopathological assessment for tumors of the sellar region was mixed gangliocytoma-pituitary adenoma, representing 77.8% of all intracranial cases. As for the extracranial tumors, the majority of them originated in the lung and pancreas (50% and 35%, respectively), with typical bronchial carcinoid as the most common histopathological diagnosis. Albeit rare, GHRH secretion in other tumors, including pheochromocytoma, lymphoma, paraganglioma, or thymoma has also been reported (4, 11, 15, 34–37). In some cases, tumor resection preceded possible acromegaly development (37–39). According to gross pathology data available for 72 patients, extracranial tumors measured 6.6 cm on average, ranging from 1 to 25 cm. Consequently, most of them were apparent in conventional imaging, with an estimated 86% sensitivity of computed tomography (CT) scan described in the French series of 21 cases (40). The largest pituitary mass found in a case of a mixed gangliocytoma-pituitary adenoma measured 6.5 cm (41). In a few cases, multiple pancreatic tumors were identified (13, 42–44). Gangliocytomas may present a characteristic MRI appearance. Usually these tumors consist of cystic and solid components. Gangliocytomas are hypo- to isointense relative to cortex on T1-weighted images and hyperintense on T2/FLAIR images. The solid portions of the tumors show variable enhancement after gadolinium administration: from none to striking homogeneous enhancement. About one-third of cases contain calcifications, which can be seen on susceptibility weighted imaging (SWI) sequence as low signal structures. There are no signs of hemorrhage or necrosis within the tumor, which may be present in common somatotropinomas. Gangliocytomas can occur anywhere in the central nervous system, however the most common and typical location is the temporal lobe. Other reported sites include the brainstem, sellar region, and spinal cord. At diagnosis, lymph node or distant metastases were present in 42 patients with liver, bones, and lung as the most frequent metastatic site. Presence of metastatic cancerous cells was also reported in the breast, spleen, central nervous system, or heart tissue in isolated cases (33, 40, 45–48). Since lung and pancreas tumors constituted the majority of GHRH-secreting tumors, an additional comparison of their main features and treatment results has been performed in **Table 3**, indicating that pancreatic tumors were associated with multiple endocrine neoplasia 1 (MEN 1) syndrome in over half of the cases and were more inclined to produce other hormones, especially insulin, gastrin, and pancreatic polypeptide (40, 42, 44, 49–52).

**Table 2:** Origin of the tumor.

Tumor site (n = 127)		n	%
**Intracranial (n = 27)**			
Sellar region		27	100%
**Extracranial (n = 100)**			
Lung (n = 50)	Typical bronchial carcinoid	43	43%
	Atypical bronchial carcinoid	4	4%
	Other	2	2%
	Lack of histopathological examination	1	1%
Pancreas		35	35%
Gastrointestinal tract		5	5%
Adrenal gland		3	3%
Thymus		2	2%
Liver		1	1%
Mediastinum		1	1%
Retroperitoneum		1	1%
Unidentified		2	2%

**Table 3:** Lung and pancreatic tumor comparison.

		Lung (*n = 50*)	Pancreas (*n = 35*)
Age at diagnosis (years)	Median (range)	42 (19-77)	37 (14-67)
	Mean ± SD	43 ± 14.3	40 ± 13.9
Diagnostic delay (years)	Median (range)	8 (2-22)	6 (2-19)
	Mean ± SD	8.5 ± 6	7.1 ± 4.8
Tumor size (cm)	Median (range)	5.9 (1.2-10)	6 (1-25)
	Mean ± SD	5.5 ± 2.5	6.7 ± 5
Hyperprolactinemia (%)		30%	57%
Other hormones (%)*	Serum elevated levels	42%	69%
	Immunohistochemistry	8%	40%
Metastases (%)		42%	37%
MEN 1 syndrome		0	18
Remission (full or partial) (%)		78%	69%
Survival rate (%)		94%	86%

*Other hormones include: adrenaline, adrenocorticotropic hormone (ACTH), calcitonin, cortisol, follicle-stimulating hormone (FSH), glucagon, gastrin, insulin, luteinizing hormone (LH), noradrenaline, pancreatic polypeptide (PP), parathormone (PTH), serotonin, somatostatin, thyroid-stimulating hormone (TSH), triiodothyronine (T3) and thyroxine (T4), vasoactive intestinal peptide (VIP).

### Clinical Presentation

Overt acromegaly at different stages was presented by 124 patients, ranging from mild acral enlargement to advanced metabolic complications such as hypertension, diabetes, or hyperparathyroidism, considerably reducing the quality of life (53). Acromegaly symptoms did not differ from those of the classic form of the disease (30, 31, 54). In almost half of the cases, elevated levels of other hormones were observed (**Table 3**). Hyperprolactinemia was the most common symptom associated endocrinopathy which was documented in 44 patients (34.7%) and though usually asymptomatic, it manifested with amenorrhea-galactorrhea syndrome in some patients (36, 55–62). Prolactin hypersecretion derived rather from GHRH-induced pituitary hyperplasia or stalk compression than from the ectopic tumor itself, as its expression was documented immunohistochemically only in intracranial tumor cases. Elevated prolactin levels occur more often in patients with acromegaly caused by an ectopic GHRH source than in those with pituitary acromegaly (30). Less common manifestations included diabetes insipidus (63), Zollinger-Ellison syndrome (49, 51), Cushing syndrome (37, 59, 64), carcinoid syndrome (55), and typical pheochromocytoma symptoms (34), as appropriate for corresponding tumors. MEN 1 syndrome was highly probable in 23 patients based on clinical features. Genetic confirmation of MEN1 mutation was available for 19 tumors, including 18 pancreatic NETs and one thymic carcinoid (13, 36, 40, 42–44, 52, 57, 61, 65, 66). In 4 patients, genetic testing had not been performed. Apart from acromegaly and other features caused by pancreatic tumors, the syndromes included hyperparathyroidism in almost all MEN1 cases (13, 36, 40, 42–44, 52, 57, 61, 65), as well as gonadotroph (42) or mixed PRL-GH (40) secreting pituitary adenomas. A null cell pituitary tumor was also detected in one case (65). Some patients suffered from visual field disturbances and severe headaches due to large masses located in the sellar region, mostly macroadenoma or somatotroph hyperplasia (41, 47, 59, 67–72). In 3 cases, acromegaly signs have not been observed despite elevated growth hormone levels (37–39). Taking the typical long course of acromegaly into consideration, it is possible that the disease was diagnosed in its initial stages based on hormone and imaging results, enabling prompt and effective treatment. In some patients, acromegaly symptoms manifested for a long time after surgical treatment of an initially asymptomatic bronchial or pancreatic tumor, due to relapse or remaining metastasis. The longest latency period in these cases lasted as many as 30 years (42, 43, 45, 47, 48, 73–75).

### Diagnosis of Ectopic Acromegaly

The most described symptom in cases at preliminary diagnosis was acromegaly, recognized by clinical signs and confirmed with GH level not suppressed after oral glucose tolerance test (OGTT) and/or elevated insulin-like growth factor 1 (IGF-1) concentration. Ectopic source should be suspected when no improvement is observed after pituitary surgery, which was the case in 21 patients (17, 19, 35, 40, 42, 56, 57, 61, 66, 73, 76–82). Other findings leading to ectopic GHRH secretion diagnosis were acromegalic features without any pituitary lesion in magnetic resonance imaging or conversely, acromegaly in the setting of a previously known non-pituitary tumor, sometimes accompanied by other manifestations mentioned above (20.5% and 39.3% of the patients, respectively). In 24 cases of intracranial tumors, ectopic acromegaly was not suspected until the histopathological examination revealed unphysiological expression of GHRH, mostly by gangliocytoma cells (6). Diagnostic criteria of acromegaly, due to GHRH-producing tumor, are met when hypersecretion of GHRH is substantiated in a patient with overt acromegaly and recovery following the resection of corresponding tumor is observed (3, 7). GHRH tumoral secretion can be proved by measuring its plasmatic levels and by positive immunostaining or radioimmunoassay with antibodies anti-GHRH 1-40 and anti-GHRH 1-44 in histopathological examination (3, 83). However, though useful,the immunohistochemical technique is not widely available due to limited access to reagents and tumor tissue which may not always be obtained in the required amount (19). Other less common laboratory methods include hormone extraction (50, 56, 76), measurement of arterio-venous gradient of GHRH across tumoral tissue (57, 84), high performance liquid chromatography (HPLC), or ion exchange chromatography (73, 85) and GHRH mRNA detection with polymerase chain reaction (PCR) (63, 86, 87). Before GHRH isolation in 1982, its possible secretion was tested mostly with bioassay, which is an indirect examination based on GH production by rat pituitary cells triggered by the substance obtained from tumor extracts (88). This method may still serve as an additional test and was recently performed on cultured human pituitary cells (16, 60). As shown in **Table 4**, GHRH expression was documented histopathologically in 99 tumors, mostly by means of immunostaining which was performed in 83 cases. Although GHRH expression by various tumors may be considered more often than it was initially believed, only a small number of patients present overt clinical acromegaly. *In vitro* tests with immunohistochemistry (IHC) and radioimmunoassay (RIA) using anti-GHRH antibodies revealed GHRH expression in up to 14% and 56% (IHC and RIA, respectively) of all type tumors, mostly small cell lung carcinoma, breast carcinoma, and pheochromocytoma, however, acromegalic features were rarely observed (8, 30, 89). This may be due to different reasons, beginning with the possibility that produced GHRH is either not released from the tumor or its amount is not enough to stimulate pituitary cells to produce GH. Moreover, ectopic GHRH activity might be reduced due to abnormal chemical structure or concurrent secretion of somatostatin from tumoral tissue (4, 81). Positive immunostaining for hormones other than GHRH was documented in 47 neoplasms; 23 of them were intracranial tumors, mostly mixed gangliocytoma-pituitary adenoma positive for GH and sometimes also PRL. Somatostatin expression has been found in both intra- and extracranial tumor tissues (6 and 9 documented cases, respectively). Other marked substances included ACTH, calcitonin, insulin, gastrin, glucagon, PP, serotonin, and VIP, produced one or more at a time by several tumors (16, 33, 40, 77, 90). As above mentioned, tumoral hormone expression has not always translated to elevated plasmatic levels, let alone the symptoms.

**Table 4:** Diagnostic methods used to confirm GHRH-induced acromegaly.

	n	%
GHRH plasma concentration	84	66.1%
Histopathological confirmation of GHRH production	99	78%
Both methods	53	41.7%

### Hormonal Evaluation

GHRH plasma levels were available for 84 patients with a median concentration of 1,273 ng/L, as shown in **Table 5**, along with other growth hormone axis levels. The cutoff suggesting ectopic acromegaly (triggered by GHRH ectopic secretion) is a GHRH level of 300 ng/L according to Scheithauer et al. (91). However, in 2012 Garby et al. proposed the threshold of 250 ng/L as a highly specific marker of an ectopic release of GHRH causing acromegaly based on a series of 21 cases (40). In reviewed cases, 76 out of 81 patients with data available in SI values (93.8%) had GHRH levels above the 250 ng/L cutoff value. Elevated GHRH plasma level (especially >250-300 ng/L), may enable differentiation between ectopic and eutopic acromegaly which usually presents with undetectable GHRH serum concentration (10). Interestingly, in patients with hypothalamic GHRH-secreting tumors, plasma level of this hormone is also low (92, 93). The same laboratory criteria are used to diagnose both pituitary and ectopic acromegaly, namely GH hypersecretion, lack of suppression of GH levels during oral glucose tolerance test (OGTT), and elevated IGF-1 levels (3, 7) and values are shown in **Table 5**. It has been described that ectopic acromegaly is more frequently related with paradoxical serum GH rise (>50% of baseline) after TRH or glucose administration. Another noticed difference was that after GHRH administration, GH rise was attenuated in ectopic acromegaly (7, 30). The most popular commercially available GHRH tests are the enzyme-linked immunosorbent assays (ELISA), which use highly specific antibody-antigen interactions. There are GHRH tests with different sensitivities available for various types of samples. ELISA is the gold standard of immunoassays. Another sensitive but less commercial method of quantifying GHRH in liquid sample is RIA, a method based on radiolabeled antibodies (I^125^). Some examples for the most commercially available ELISA kits are: Human GHRH ELISA Kit (sandwich ELISA), detecting GHRH in plasma, tissue homogenates, and other biology fluids, with sensitivity < 0.75 pg/mL (Antibodies.com8, United Kingdom; Biorbyt, United Kingdom). Human GHRH ELISA Kit (sandwich ELISA), detecting GHRH in serum, plasma and other biological fluids with sensivity 9.38 pg/mL (Novus Biologicals, LLC, USA; LSBio, USA; Assaygenie, Ireland). Human GHRH ELISA Kit (sandwich ELISA), detecting GHRH in serum, plasma, cell culture supernatants, body fluid, and tissue homogenate with sensitivity 1.0 pg/mL (MyBioSource, Inc.USA). Taking into consideration the poor accessibility of GHRH plasma measurement in some areas of the world, the most common side effect, and relatively large dimensions of the related extracranial tumors, the authors suggest that a chest radiograph and an abdomen ultrasound should be performed in every acromegaly case as a minimum diagnostic procedures in order not to overlook a source of an ectopic production. In some cases abdomen and chest CT should be considered, especially in cases of unequivocal acromegaly diagnosis and the absence of a pituitary tumor in MR imaging.

**Table 5:** Serum/plasma concentrations of hormones in the hypothalamic–pituitary–somatotropic axis (HPS axis) in patients with ectopic acromegaly.

		*n*	Mean ± SD	Median (range)
GHRH (ng/L)		81	8,965 ± 19,547	1,273 (60.1-145,000)
GH (ng/mL)	Basal	94	54.2 ± 83.7	29.6 (1.7-488)
	OGTT Nadir	66	43.1 ± 60.6	25.4 (0.1-323)
	Maximum value in TRH test	31	178 ± 272	66.5 (6.9-1,264)
	Post GHRH test	7	33.2 ± 21.9	33.2 (17.7-487)
IGF-1*		84	2.7 ± 1.5	2.4 (1-11.73)

*expressed as patient’s IGF-1 value/ULN (upper limit of normal).

### Pituitary Morphology

Pituitary imaging was available for 114 patients (89.8%), with MR imaging being the most commonly used method. As shown in **Tables 6**, **7**, its interpretation might be difficult and misleading in GHRH-induced acromegaly. Exposure to GHRH hypersecretion often leads to pituitary somatotroph hyperplasia, which is considered to be a characteristic feature of ectopic acromegaly. Apart from this, somatotroph hyperplasia has only been found in patients with McCune-Albright syndrome (4). However, studies show that pituitary adenoma may also occur in the course of ectopic GHRH secretion when the exposure is prolonged which was proved in laboratory transgenic mice (94). Although rare, such cases have also been described in patients, however it has not been proved that the adenoma was due to GHRH secretion and not incidental (40, 65, 77). The association has also been shown for sellar region gangliocytoma where the proximity of GHRH source may result in paracrine stimulation of pituitary cells eventually leading to adenomatous transformation (41, 62, 95). It has been suggested that somatotroph transformation in response to ectopic GHRH may exhibit a continuum model of transformation rather than a surge character of changes and therefore both hyperplasia and adenomatous cells may be present at the same time (57, 96, 97). Hence, proper distinction between pituitary adenoma and hyperplasia is not always achievable by means of imaging methods. This may, in consequence, lead to unnecessary surgical resection, especially when there is no other indication of an extracranial neoplasm as an underlying cause of acromegaly. Pituitary surgery *via* transsphenoidal approach or craniotomy was performed in 54 patients. In 28 cases it appeared to be curative, as the pituitary tumor has been found to cause acromegaly. In an additional 5 patients it served as symptomatic treatment of bitemporal hemianopsia or headache induced by the pituitary mass. However, in 21 cases (38.9% of performed procedures), resection was proved not to be necessary, revealing hyperplasia or normal pituitary tissue in histopathological examination and no improvement afterwards. This stresses the need for a cautious approach while interpreting pituitary images and proves there is necessity for accurate hormonal evaluation, especially in ambiguous cases, in order to avoid unnecessary surgery. In patients with extracranial ectopic source of GHRH, sellar enlargement proved to be reversible in many cases and pituitary size diminished significantly after resection of the primary tumor or somatostatin receptor ligands (SRL) therapy (21, 49, 52, 58, 68, 98).

**Table 6:** Pituitary imaging and histopathology of intracranial GHRH-secreting tumors.

	*n*	%
**Imaging *(n = 18)***		
Adenoma	12	66.7%
Unclear lesion	5	27.8%
Enlargement	1	5.5%
**Histopathology *(n = 27)***		
Mixed gangliocytoma-pituitary adenoma	21	77.8%
Gangliocytoma	3	11.1%
Adenoma	1	3.7%
Lymphoma with somatotroph hyperplasia	1	3.7%
Normal	1	3.7%

**Table 7:** Pituitary imaging and histopathology of extracranial GHRH-secreting tumors.

	*n*	%
**Imaging *(n = 96)***		
Enlargement	43	44.8%
Adenoma	20	20.8%
Normal	18	18.8%
Unclear lesion	10	10.4%
Empty sella	3	3.1%
Microcystic lesion	2	2.1%
**Histopathology *(n = 29)***		
Hyperplasia	20	69%
Adenoma	3	10.3%
Mixed adenoma and hyperplasia	3	10.3%
Other	2	6.9%
Normal	1	3.5%

### Treatment Strategies and Prognosis

Despite various treatment modalities being used in ectopic acromegaly management, tumor surgical resection remains a method of choice due to its highest effectiveness and should be performed when feasible (3). Complete tumor resection was the main treatment method for 85 patients, including adenomectomy in 26 of 27 intracranial tumors cases. In a few cases, surgical resection of liver metastases was performed with satisfactory results (40). SRL therapy is the preferred treatment option for patients with inoperable tumors or disseminated metastatic disease, yet, it may also serve successfully as an adjuvant therapy for patients who undergo surgical procedures (3). Therapy with SRL was administered to 37.8% of patients at different stages of treatment, being the main method in 22 cases (9, 40, 45, 47, 50, 68, 75, 99, 100). However, in some cases it was proved to not be sufficiently effective, presumably due to the lack of somatostatin receptor 2 (SSTR-2) in tumor tissue, which is also uncommon, in hyperplastic somatotroph cells (61). In other cases, even if somatostatin receptors were not found in tumor tissue, SRL therapy showed at least partial efficacy due to typical SSTR expression in somatotroph cells (61). In the other cases it appeared to be the only successful method even after surgical treatment (78). The efficacy of surgical resection and SRL therapy cannot be righteously compared in this condition, as the second method has usually served as basic treatment in more advanced cases, often with metastatic disease which itself is a factor of worse prognosis (36). Along with SRL, other supportive methods that may be mentioned are chemotherapy, radiotherapy, immunotherapy, metastases embolization, and other hormonal treatments such as bromocriptine or pegvisomant (9, 18, 33, 36, 40, 42, 43, 46, 49, 51, 73, 78, 90, 96). Altogether, 41 patients received one of these adjuvant treatment options, with pituitary radiation being the most frequent modality (18, 19, 40, 59, 64, 67, 70, 77, 78, 96). Therapeutic results were available for 115 patients with an overall survival rate of 88.7%. Complete and partial remission following treatment was documented in 47.8% and 28.7%, respectively. As for patients with metastatic tumors, their overall survival rate did not differ much from patients with non-metastatic tumors, with outcomes of 83% and 92%, respectively. Nevertheless, metastatic disease appears to be a factor of treatment with worse results, as full or partial recovery was documented for 87.8% of patients without metastases and only 56.1% with disseminated tumors. Relapse and progression were also observed much more frequently within the latter (40, 57, 91). Mean follow-up was 4.2 ± 5.2 years for all 81 patients with available data. This outcome is in accordance with the literature data indicating that prognosis in neuroendocrine tumors is rather favorable even if diagnosed at a metastatic stage and the course of disease remains indolent in most cases (101, 102). Recovery was defined by improvement of symptoms as well as normalization in GH and IGF-1 serum levels. Full GHRH normalization is achievable after surgical treatment but not with SRL therapy. Hormonal treatment appears to reduce GHRH level but not below the detection limit (10). Evaluation of plasma GHRH may be considered useful when anticipating relapse as the rise of GHRH concentration can occur before recurrence of clinical manifestations (11).

## Conclusion

Acromegaly caused by GHRH release is very rare disease, although one should be aware it exists. Signs, symptoms, and common hormonal evaluation do not differentiate clearly between tumors secreting GH and GHRH. Pituitary imaging does not provide proper diagnosis between pituitary adenoma and hypertrophy and could result in unnecessary surgery. GHRH tumor secretion can be proved by measuring its plasmatic levels and using histopathological methods with immunohistochemical techniques, however, these are still hardly accessible procedures. GHRH level above 250 ng/L indicates a high probability of an ectopic cause of acromegaly. Vast majority of GHRH-producing tumors are neuroendocrine tumors, quite often associated with MEN-1 syndrome.

## Author Contributions

Idea for the article: MB, MK, and KZ; literature search and data analysis: IZ and GG, drafting the manuscript: MK, IZ, and GG; supervision and critical revision of the work: MB, MK, and KZ. All authors contributed to manuscript revision, read, and approved the submitted version.

## Conflict of Interest

The authors declare that the research was conducted in the absence of any commercial or financial relationships that could be construed as a potential conflict of interest.

## Publisher’s Note

All claims expressed in this article are solely those of the authors and do not necessarily represent those of their affiliated organizations, or those of the publisher, the editors and the reviewers. Any product that may be evaluated in this article, or claim that may be made by its manufacturer, is not guaranteed or endorsed by the publisher.
